# Effects of dexamethasone on immune dysfunction and ventilator-associated pneumonia in COVID-19 acute respiratory distress syndrome: an observational study

**DOI:** 10.1186/s40560-021-00580-6

**Published:** 2021-10-18

**Authors:** Martin Cour, Marie Simon, Laurent Argaud, Guillaume Monneret, Fabienne Venet

**Affiliations:** 1 Hospices Civils de Lyon, Intensive Care Medicine, Service de Médecine Intensive-Réanimation, Hôpital Edouard Herriot, 5, Place d’Arsonval, F-69437 Lyon Cedex 03, France; 2 Faculté de médecine Lyon-Est, Université de Lyon, Université Claude Bernard Lyon 1, F-69437 Lyon, France; 3 Immunology Laboratory, Hospices Civils de Lyon, Edouard Herriot Hospital, 69437 Lyon, France; 4 Centre International de Recherche en Infectiologie (CIRI), Inserm U1111, CNRS, UMR5308, Ecole Normale Supérieure de Lyon, Université Claude, Bernard-Lyon 1, Lyon, France

**Keywords:** SARS-CoV-2, Monocyte HLA-DR, CD4 + lymphocyte, Nosocomial infection, Intensive care unit

## Abstract

Dexamethasone improves survival of patients with COVID-19 acute respiratory distress syndrome, but might shorten the delay between the start of invasive mechanical ventilation and the occurrence of ventilator-associated pneumonia, suggesting possible worsening of COVID-19-induced immune dysfunction with this treatment. In a prospective observational study, we found that mechanically ventilated patients with COVID-19 treated with dexamethasone presented earlier ventilator-associated pneumonia, had significantly lower monocyte Human Leukocyte Antigen-DR expression and number of circulating CD4 + cells after ICU admission, than those not treated with corticoids.

Following the RECOVERY trial, dexamethasone became a standard treatment for most COVID-19 patients with acute respiratory distress syndrome (ARDS) [1]. Indeed, low dose of dexamethasone significantly decreased day-28 mortality in COVID-19 ARDS [1]. This benefit is thought to be related to the anti-inflammatory properties of the drug. However, concerns raised about the risk of nosocomial infection [2]. A large multicentric observational study reported that ventilator-associated pneumoniae (VAP) occurred significantly earlier (but not more often) in patients treated with dexamethasone than in those who were not [3], suggesting a possible worsening of immune dysfunction with this drug. Nevertheless, the effects of dexamethasone on COVID-19-induced immune dysfunction remain undetermined. We, therefore, conducted a study to compare immune profiles of COVID-19 patients admitted to intensive care unit (ICU) for ARDS and VAP characteristics before and after systematic use of dexamethasone.

This project was part of the prospective observational study REA-IMMUNO-COVID (RICO) that was approved by an institutional ethics committee (N°IRB/IORG: #IORG0009918) and registered (ClinicalTrials.gov: NCT04392401) [4].

Adult patients with COVID-19 ARDS (defined by Berlin criteria) hospitalized in a French academic ICU for ≥ 7 days during the first wave (W1, march–April 2020) or the second wave (W2, October–November 2020) of the pandemic were screened and included if they (1) were included in RICO, (2) were not immunocompromised (i.e. no organ transplantation, HIV, cancer and/or chronic treatment with immunosuppressive drugs), and (3) had no missing data of interest. In addition, during W1, patients were excluded if they received corticoids before the first assessment of immune function. During W2, dexamethasone was given intravenously at a dose of 6 mg/day for 10 days, as in RECOVERY [1] with no other significant therapeutic changes versus W1. VAP was defined according to international guidelines as a microbiologically confirmed pneumonia diagnosed after 48 h of intubation [5]. Monocyte Human Leukocyte Antigen-DR (mHLA-DR) expression, absolute lymphocyte count and number of circulating CD4 + cells were assessed within the first 48 h of ICU admission (D1), 72–96 h after admission (D3) and between days 7 and 9 (D7). We also recorded highest body temperatures of the day at D1, D3 and D7, as hyperthermia has been shown to be associated with mortality [6] and could be dampened by dexamethasone.

Among the 68 patients who were screened (W1: *n* = 31; W2: *n* = 37), 36 (53%) were included in the present study (W1: *n* = 18; W2: *n* = 18). The reasons for non-inclusion were: not being included in RICO (*n* = 24), immunocompromised status (*n* = 6) and missing data (*n* = 2). No patient received immunosuppressive drugs other than corticosteroids (e.g., tocilizumab) or remdesivir. Baseline patients’ characteristics did not differ between the 2 waves (Table 1). During W2, in all patients but one, dexamethasone was started the day of ICU admission. The *Sequential Organ Failure Assessment* (SOFA) score at D1 was significantly higher in W1 versus W2 (Table 1). At D1, highest body temperature, mHLA-DR and CD4 + lymphocytes, were significantly lower in patients treated with dexamethasone compared to those who were not (Fig. 1A, B and D). In both groups, median values of mHLA-DR (Fig. 1B) and lymphocyte counts (Fig. 1C and D) remained below normal values over 7 days. Afterwards, these differences faded. The proportion of patients who developed VAP was similar in the 2 groups. However, compared to W1, VAP occurred significantly earlier, duration of mechanical ventilation was significantly shorter and day-90 mortality lower during W2 (Table 1).

**Table 1:** Patients characteristics and outcomes

	Wave 1 (*n* = 18)	Wave 2 (*n* = 18)	*P*
Age—years	66 [61–70]	70 [61–73]	0.419
Male gender—*n* (%)	14 (78)	15 (83)	> 0.99
Body mass index—kg/m^2^	31 [28–36]	31 [28–36]	0.567
Hypertension—*n* (%)	8 (44)	10 (56)	0.740
Diabetes—*n* (%)	5 (63)	9 (50)	0.310
SAPS II score—points	39 [33–50]	37 [31–47]	0.486
SOFA score at Day 1^a^—points	8 [6–9]	5 [2–8]	0.041
Delay between symptoms and ICU admission—days	6 [4–9]	9 [7–11]	0.09
Delay between ICU admission and intubation—days	1 [0–2]	1 [1, 2]	0.356
Bacterial superinfection at intubation—*n* (%)	6 (33)	3 (17)	0.440
Lowest PaO_2_/FiO_2_ ratio during ICU stay—mmHg	83 [62–112]	81 [62–100]	0.784
Severity of ARDS—*n* (%)			0.460
Moderate	6 (33)	4 (22)	
Severe	12 (67)	14 (78)	
Duration of invasive mechanical ventilation—days	29 [18–47]	12 [8–22]	< 0.01
Ventilation acquired pneumoniae—*n* (%)	15 (83)	13 (72)	0.802
Delay between intubation and VAP—days	13 [8–27]	7 [5–11]	0.020
Day 28 mortality—*n* (%)	5 (28)	2 (11)	0.210
Day 90 mortality—*n* (%)	8 (44)	3 (17)	0.150

*SAPS II* Simplified Acute Physiology Score II, *SOFA* Sequential Organ Failure Assessment, *ICU* intensive care unit, *ARDS* acute respiratory distress syndrome, *VAP* ventilation acquired pneumoniae. Results were compared using Mann–Whitney test or Fisher’s exact test, as appropriate

^a^Day 1: day of the first measures of lymphocytes counts and monocyte HLA-DR expression

Data are expressed as median (interquartile) and number (percentage)

**Fig. 1:**
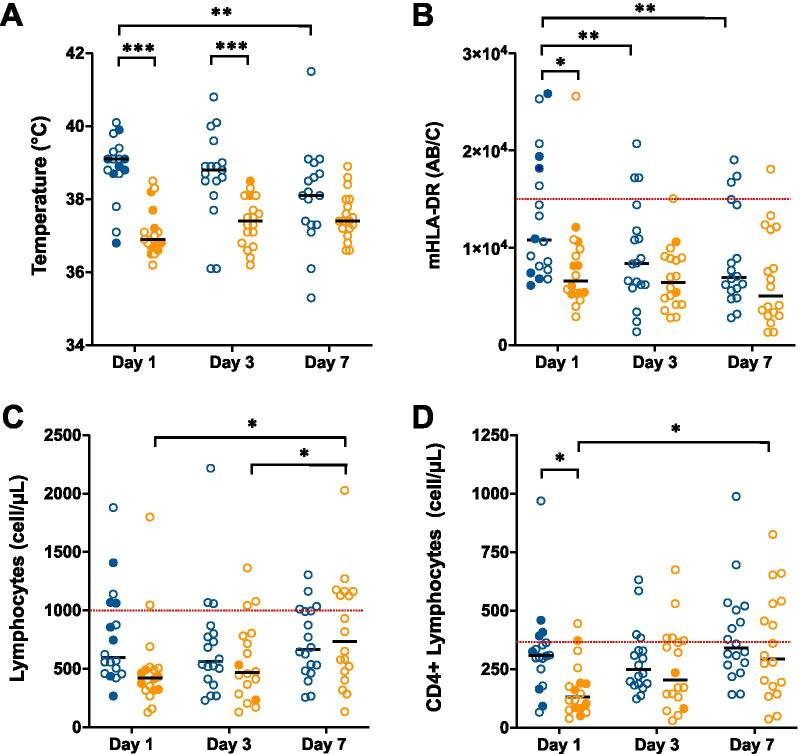
Temperature, monocyte HLA-DR expression and lymphocytes count over time in COVID-19 patients with ARDS treated or not with dexamethasone. Individual (circles) and median (black hyphen) values of highest body temperature of the day (**A**), monocyte HLA-DR expression (mHLA-DR) expressed as numbers of antibodies bound per cells (AB/C), **B**), total lymphocytes count (**C**), and number of CD4 + lymphocytes (**D**) are reported at day 1, 3, 7 after admission for 18 patients with COVID-19-induced with ARDS during the first wave of the pandemic (blue circle) who did not receive dexamethasone and for 18 patients of the second wave treated with dexamethasone (orange circle). Empty circles indicate patients receiving invasive mechanical ventilation and full circles patients not intubated. Red dashed line indicates lower normal values for mHLA-DR and lymphocytes counts. *p < 0.05; **p < 0.01; ***p < 0.001 in two-way ANOVA with repeated measures

This study confirms the profound immunosuppression previously described in critically ill COVID-19 patients [4]. Compared to W1, a low dose of dexamethasone was associated with worsened immune dysfunction at D1 (as assessed by mHLA-DR and CD4 + lymphocytes), earlier occurrence of VAP but also with prevention of fever and shorter duration of mechanical ventilation. The worsening of the immune dysfunction in patients from W2 could not be explained by baseline characteristics including organ dysfunctions as assessed by the SOFA score that were similar between the 2 groups.

Data on effects of corticoids on immune profile in critically ill patients remain scarce. In septic shock, low dose hydrocortisone was reported to slightly decrease mHLA-DR while preventing release of pro-inflammatory cytokines [7]. In the present study, dexamethasone also dampened inflammation as shown by the dramatic decrease in fever observed during W2.

Even if incidence of VAP did not differ before and after systematic use of dexamethasone, this complication occurred significantly earlier during W2. A similar result was recently reported in a large multicentric observational study comparing VAP during W1 and W2 [3]. However, the association between early nosocomial infection and putative dexamethasone-induced immunodepression seems to be advantageously balanced by a shorter exposition to invasive devices in patients with COVID-19 ARDS. Despite a limited number of highly selected participants and possible changes in the management of patients over time that may limit generalizability, our results should alert physicians to the high rate of early VAP in COVID-19 ARDS patients treated with dexamethasone and to the necessity of a carefully immune monitoring of these patients.

## Data Availability

All data generated or analyzed during this study are included in this published article (and its supplementary information files).

## Abbreviations

ARDS: Acute respiratory distress syndrome
ICU: Intensive care unit
mHLA-DR: Monocyte human leucocyte antigen-DR
VAP: Ventilator-associated pneumoniae
W1: Wave 1 of the COVID-19 pandemic
W2: Wave 2 of the COVID-19 pandemic
